# Phylogenetic tree information aids supervised learning for predicting protein-protein interaction based on distance matrices

**DOI:** 10.1186/1471-2105-8-6

**Published:** 2007-01-09

**Authors:** Roger A Craig, Li Liao

**Affiliations:** 1 Department of Computer & Information Sciences, University of Delaware, Newark, DE 19716, USA

## Abstract

**Background:**

Protein-protein interactions are critical for cellular functions. Recently developed computational approaches for predicting protein-protein interactions utilize co-evolutionary information of the interacting partners, e.g., correlations between distance matrices, where each matrix stores the pairwise distances between a protein and its orthologs from a group of reference genomes.

**Results:**

We proposed a novel, simple method to account for some of the intra-matrix correlations in improving the prediction accuracy. Specifically, the phylogenetic species tree of the reference genomes is used as a guide tree for hierarchical clustering of the orthologous proteins. The distances between these clusters, derived from the original pairwise distance matrix using the Neighbor Joining algorithm, form intermediate distance matrices, which are then transformed and concatenated into a super phylogenetic vector. A support vector machine is trained and tested on pairs of proteins, represented as super phylogenetic vectors, whose interactions are known. The performance, measured as ROC score in cross validation experiments, shows significant improvement of our method (ROC score 0.8446) over that of using Pearson correlations (0.6587).

**Conclusion:**

We have shown that the phylogenetic tree can be used as a guide to extract intra-matrix correlations in the distance matrices of orthologous proteins, where these correlations are represented as intermediate distance matrices of the ancestral orthologous proteins. Both the unsupervised and supervised learning paradigms benefit from the explicit inclusion of these intermediate distance matrices, and particularly so in the latter case, which offers a better balance between sensitivity and specificity in the prediction of protein-protein interactions.

## Background

Protein-protein interactions play a key role in cellular functions, and thus, to complement the experimental approaches [1,2], many computational methods have recently been developed in systems biology for predicting whether two proteins interact, based on what is already known about these proteins. One type of data used for prediction is the phylogenetic profile of a protein – a string of ones and zeros encoding respectively the presence and absence of the protein in a group of genomes, conserved operons, gene fusions, etc. [3-6]. The rationale is that interacting proteins tend to co-evolve, and therefore should have similar phylogenetic profiles. Recently, to enhance the prediction accuracy, the focus has been given to using the similarity of phylogenetic trees to infer interactions between receptors and ligands [6-8].

Of particular interest is the so-called mirror tree method by Pazos and Valencia [6]. The mirror tree method predicts protein-protein interactions under the assumption that the interacting proteins show similarity in the molecular phylogenetic protein trees because of the co-evolution caused by the interaction. However, it is difficult to directly evaluate the similarity between a pair of molecular phylogenetic trees. Instead, the mirror tree method compares a pair of distance matrices by calculating the Pearson correlation coefficient for the corresponding elements in the two matrices, and uses the correlation coefficient as a measure to evaluate the extent of co-evolutionary behavior between two proteins.

To address the issue of high rate of false positives with the mirror tree method, recently, Sato *et al *[9] suggested that the information about the phylogenetic relationships of the host genomes be excluded by a projection operation, and only the residual information in the distance matrices be used for the calculation of the correlation coefficient between proteins. As a result, significant improvement in prediction specificity was achieved, though at a cost of losing some sensitivity. A similar yet more sophisticated approach is proposed in Pazos *et al *[10] to correct the distance matrices based on the phylogenetic tree, which incorporates information on the overall evolutionary histories of the species (i.e., the canonical "tree of life"). In addition to adjusting the distance matrices by excluding the expected background similarity due to the underlying speciation events, this tree of life mirror tree (*tol-mirrortree*) method can also detect non-canonical evolutionary events, in particular horizontal gene transfers. While both Pazos *et al*'s *tol-mirrortree *method and Sato *et al's *projection approach are concerned with – and quite successful at – removing some background from the inter-matrix correlation, like the original mirror tree method they do not directly address the intra-matrix correlations, which can be informative and critical in revealing co-evolution. For example, in some recent related studies, the columns and rows of the distance matrices are reshuffled in an attempt to discover maximal similarity between two matrices in order to predict interaction specificity when paralogs are involved [11-13].

In this work, we propose a novel, simple method to extract the intra-matrix correlational information with reference to the species tree of the host genomes and to represent said information in a way that is conducive to a supervised learning paradigm. We tested our method on the same dataset used in [9], which consists of interacting proteins from *E. coli*, where these interactions are experimentally verified. The results from a series of leave-one-out cross validation experiments showed that the prediction accuracy was greatly increased with our data representation method.

## Methods

### Dataset

We selected the same data set as used in [9], so that the performance of the different methods can be compared. The 13 pairs of interacting proteins are from *E. coli*, and the interaction within each pair has been experimentally verified, as documented in the Database of Interacting Proteins (DIP) [14], and no interaction outside the pairing is known. So, these 26 proteins make up 26 × 25/2 = 325 distinct pairs but only 13 of them contain truly interacting partners. For each of these 26 proteins, its putative orthologs from 41 bacterial genomes are selected from KEGG/KO database [15], and a 41 × 41 distance matrix is constructed, giving the genetic distance between any pair of these 41 orthologs. The genetic distances were calculated using the PROTDIST module in the PHYLIP package [16] and the score table by Jones, Taylor, and Thornton [17], from a multiple alignment of these 41 orthologous proteins, constructed using MAFFT [18] software. The 13 pairs of proteins and the 41 source organisms are listed in Tables 1 and 2 respectively.

**Table 1:** The 13 pairs of interacting proteins in E. coli

**Interacting pairs**		
1	sucC	sucD
2	atpA	atpD
3	rpoA	rpoB
4	secA	secY
5	carA	carB
6	ruvA	ruvB
7	iscS	iscU
8	dnaE	dnaN
9	trpA	trpB
10	tufB	tsf
11	dnaA	dnaB
12	grpE	dnaK
13	clpX	clpP

**Table 2:** The 41 source organisms used to find orthologous proteins

Escherichia coli K-12 MG1655
Salmonella typhi CT1 8
Samonella enerica
Samonella typhimurium LT2
Yersinia pestis CO92
Yersinia pseudotuberculosis
Shigella flexneri 301 (serotype 2a)
Photorhabdus luminescens
Pasteurella multocida
Mannheimia succiniciproducens
Vibrio cholerae
Vibrio vulnificus YJ016
Vibrio parahaemolyticus
Photobacterium profundum
Pseudomonas aeruginosa
Pseudomonas putida
Pseudomonas syringae pv. Tomato
Acinetobacter sp. ADP1
Shewanella oneidensis
Neisseria meningitides MC58 (serogroup B)
Chromobacterium violaceum
Ralstonia solanacearum
Bordetella pertussis
Bordetella parapertussis
Nitrosomonas europaea
Campylobacter jejuni NCTC11168
Rhodopseudomonas palustris
Bacillus subtilis
Bacillus halodurans
Bacillus anthracis Ames
Bacillus cereus ATCC 14579
Bacillus thuringiensis
Bacillus lucheniformis ATCC 14580
Oceanobacillus iheyensis
Staphylococcus aureus N315 (MRSA)
Staphylococcus epidermidis ATCC12228
Corynebacterium efficiens
Streptomyces avermitilis
Propionibacterium acnes
Rhodopirellula baltica (Pirellula sp.)
Anabaena sp. PCC7120 (Nostoc sp. PCC7120)

The phylogenetic tree for these 41 reference bacterial genomes was built from the 16S rRNA sequences using the neighbor-joining module in PHYLIP package. The 16S rRNA sequences were downloaded from the KEGG/GENES database [15] and the Ribosomal Database Project-II Release 9 [19].

### Phylogenetic vectors and correlations

The original mirror tree method is proposed by Pazos and Valencia [6] to infer protein-protein interaction from correlated evolutions. The hypothesis is that two proteins should have a higher chance to share correlated evolutionary history if they interact with each other than if they do not. As the evolutionary history for a protein can be represented as a phylogenetic tree (let's call it a protein tree to distinguish from the species tree), it makes sense to compare the two protein trees to reveal any correlation between their evolutionary history. Instead of comparing two trees directly, which is a highly nontrivial task in terms of both algorithmic implementation and biological interpretation, the mirror tree method uses as a surrogate the distance matrices that store the genetic distance between the protein and its orthologs in a group of genomes. It is from these distance matrices that the proteins trees are typically reconstructed using well known algorithms such as Neighbor-Joining [20]. For two proteins A and B, the mirror tree method compares their distance matrices D_A _and D_B_, by examining how the corresponding elements are correlated. Because the distance matrices are symmetric, only the elements in the upper (or lower) triangle of the matrices are needed to calculate the correlation, which is measured as the Pearson correlation coefficient *ρ *as defined below:

ρAB=∑i=1n−1∑j=i+1n(DA(i,j)−Ave(DA))(DB(i,j)−Ave(DB))Var(DA)Var(DB)     (1)
 MathType@MTEF@5@5@+=feaafiart1ev1aaatCvAUfKttLearuWrP9MDH5MBPbIqV92AaeXatLxBI9gBaebbnrfifHhDYfgasaacH8akY=wiFfYdH8Gipec8Eeeu0xXdbba9frFj0=OqFfea0dXdd9vqai=hGuQ8kuc9pgc9s8qqaq=dirpe0xb9q8qiLsFr0=vr0=vr0dc8meaabaqaciaacaGaaeqabaqabeGadaaakeaaiiGacqWFbpGCdaWgaaWcbaGaeeyqaeKaeeOqaieabeaakiabg2da9maalaaabaWaaabmaeaadaaeWaqaaiabcIcaOiabdseaenaaBaaaleaacqqGbbqqaeqaaOGaeiikaGIaemyAaKMaeiilaWIaemOAaOMaeiykaKIaeyOeI0IaeeyqaeKaeeODayNaeeyzauMaeiikaGIaemiraq0aaSbaaSqaaiabbgeabbqabaGccqGGPaqkcqGGPaqkcqGGOaakcqWGebardaWgaaWcbaGaeeOqaieabeaakiabcIcaOiabdMgaPjabcYcaSiabdQgaQjabcMcaPiabgkHiTiabbgeabjabbAha2jabbwgaLjabcIcaOiabdseaenaaBaaaleaacqqGcbGqaeqaaOGaeiykaKIaeiykaKcaleaacqWGQbGAcqGH9aqpcqWGPbqAcqGHRaWkcqaIXaqmaeaacqWGUbGBa0GaeyyeIuoaaSqaaiabdMgaPjabg2da9iabigdaXaqaaiabd6gaUjabgkHiTiabigdaXaqdcqGHris5aaGcbaWaaOaaaeaacqqGwbGvcqqGHbqycqqGYbGCcqGGOaakcqWGebardaWgaaWcbaGaeeyqaeeabeaakiabcMcaPiabbAfawjabbggaHjabbkhaYjabcIcaOiabdseaenaaBaaaleaacqqGcbGqaeqaaOGaeiykaKcaleqaaaaakiaaxMaacaWLjaWaaeWaaeaacqaIXaqmaiaawIcacaGLPaaaaaa@7B33@

where Ave and Var represent the average and the variance of the elements in the upper triangle of a distance matrix, respectively. To apply this method for prediction, the Pearson correlation coefficient *ρ *is calculated for all distinct pairs of proteins, and these pairs are then ranked in a non decreasing order of *ρ*. With a threshold preset on *ρ*, the pairs with a higher correlation coefficient are predicted to be interacting pairs.

In two recent works [9,10], the measurement of correlations is refined by excluding the information about the phylogenetic relationships in order to overcome the problem of high rate of false positives reportedly present in the predictions using the mirror tree method. The high rate of false positives is believed to be caused by a high correlation between non-interacting proteins, which can be attributed to some common background shared by the two corresponding distance matrices, because they all are derived from orthologous proteins in the same set of *n *source organisms. That is to say, these protein trees bear some resemblance to the species tree. Sato *et al *therefore propose to exclude the species tree resemblance from the distance matrices before comparing them. Specifically, a distance matrix R is computed for the 16S rRNA sequences of these 41 genomes, from which the species tree can be reconstructed. For convenience, all the rows in the upper triangle of this 41 × 41 distance matrix are concatenated, producing a vector of dimension 820, which we refer to as |u_16s_>. Similarly, all the distance matrices for the protein trees can be transformed into a vector form, of the same dimension 820, which is termed the *phylogenetic vector*. Let |v_i_> (i = 1 to 26) be a phylogenetic vector for one of the 26 proteins in the dataset, then the resemblance of |v_i_> to |u_16s_> is measured by the projection < u_16s_|v_i _> (i.e., the inner product between |v_i_> and |u_16s_>), which is then subtracted from |v_i_>, giving a residue vector |*ε*_i_> defined as follow:

|*ε*_i_> = |v_i_> - |u_16s_> (<u_16s_| v_i _>)     (2)

Then, the Pearson correlation coefficient (is calculated for any pair of vectors |*ε*_i_> and |*ε*_j_>:

ρij=Σk=1 to 820{[|εik>- Ave(|εi>)][|εjk>- Ave(|εj>)]}/√[Var(|εi>)Var(|εj>)],     (3)
 MathType@MTEF@5@5@+=feaafiart1ev1aaatCvAUfKttLearuWrP9MDH5MBPbIqV92AaeXatLxBI9gBaebbnrfifHhDYfgasaacH8akY=wiFfYdH8Gipec8Eeeu0xXdbba9frFj0=OqFfea0dXdd9vqai=hGuQ8kuc9pgc9s8qqaq=dirpe0xb9q8qiLsFr0=vr0=vr0dc8meaabaqaciaacaGaaeqabaqabeGadaaakeaacqaHbpGCdaWgaaWcbaGaeeyAaKMaeeOAaOgabeaakiabg2da9iabfo6atnaaBaaaleaacqqGRbWAcqGH9aqpcqaIXaqmcqqGGaaicqqG0baDcqqGVbWBcqqGGaaicqaI4aaocqaIYaGmcqaIWaamaeqaaOGaei4EaSNaei4waSLaeiiFaWNaeqyTdu2aa0baaSqaaiabbMgaPbqaaiabbUgaRbaakiabg6da+iabb2caTiabbccaGiabbgeabjabbAha2jabbwgaLjabcIcaOiabcYha8jabew7aLnaaBaaaleaacqqGPbqAaeqaaOGaeyOpa4JaeiykaKIaeiyxa0Laei4waSLaeiiFaWNaeqyTdu2aa0baaSqaaiabbQgaQbqaaiabbUgaRbaakiabg6da+iabb2caTiabbccaGiabbgeabjabbAha2jabbwgaLjabcIcaOiabcYha8jabew7aLnaaBaaaleaacqqGQbGAaeqaaOGaeyOpa4JaeiykaKIaeiyxa0LaeiyFa0Naei4la8cccaGae8NgIyTaei4waSLaeeOvayLaeeyyaeMaeeOCaiNaeiikaGIaeiiFaWNaeqyTdu2aaSbaaSqaaiabbMgaPbqabaGccqGH+aGpcqGGPaqkcqqGwbGvcqqGHbqycqqGYbGCcqGGOaakcqGG8baFcqaH1oqzdaWgaaWcbaGaeeOAaOgabeaakiabg6da+iabcMcaPiabc2faDjabcYcaSiaaxMaacaWLjaWaaeWaaeaacqaIZaWmaiaawIcacaGLPaaaaaa@8F07@

where |εik>
 MathType@MTEF@5@5@+=feaafiart1ev1aaatCvAUfKttLearuWrP9MDH5MBPbIqV92AaeXatLxBI9gBaebbnrfifHhDYfgasaacH8akY=wiFfYdH8Gipec8Eeeu0xXdbba9frFj0=OqFfea0dXdd9vqai=hGuQ8kuc9pgc9s8qqaq=dirpe0xb9q8qiLsFr0=vr0=vr0dc8meaabaqaciaacaGaaeqabaqabeGadaaakeaacqGG8baFcqaH1oqzdaqhaaWcbaGaeeyAaKgabaGaee4AaSgaaOGaeyOpa4daaa@33C8@ stands for the k-th component of vector |*ε*_i_>, Ave and Var represent the average and variance of elements in a vector. It is shown in [9] that the specificity of predictions using the subtracted vectors is significantly improved, though at a cost of losing sensitivity.

In [10], phylogenetic trees (protein trees) are first reconstructed from the multiple sequence alignments of orthologous proteins using the neighbor-joining algorithm implemented in ClustalW. The protein distance matrices are then derived from these trees by summing the length of the branches connecting each pair of orthologous proteins, which are represented as tree leaves. New distance matrices for the proteins are obtained by subtracting from each value the distance between the corresponding species in the 16S rRNA distance matrix, termed as R. If we transform the matrices into vectors in the same way as used in [9], then the element-wise subtraction of 16S rRNA distance matrix R from a distance matrix P for a protein is equivalent to subtraction of two corresponding vectors,

|p'> = |p> - |r>     (2')

where |p> is the phylogenetic vector derived from the upper triangle of matrix P, and |r> is the vector from the upper triangle of matrix R. The difference between Eq(2) and Eq(2') can be seen more clearly as depicted geometrically in Figure 1. It is noted that the resulting vector |*ε*> derived from Eq(2) is guaranteed to be orthogonal to the phylogenetic tree orientation, whereas the resulting vector from Eq(2') may still have non-zero projection along the phylogenetic tree orientation, which can become minimal when the two vectors are properly rescaled using a "molecular clock" to about the same length [10]. It is also worth noting that although having phylogenetic vectors totally orthogonal to the phylogenetic tree orientation may be mathematically sound and attractive, it by no means necessarily leads to better learning and classification, as many other factors may affect the similarity between a pair of phylogenetic vectors, e.g., when there are horizontal gene transfers as pointed out and dealt with in [10].

**Figure 1:**
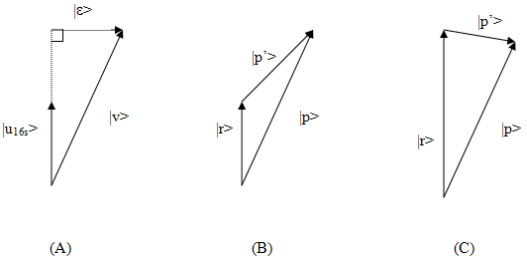
Geometric interpretation of subtracting phylogenetic background. Panel A corresponds to Eq(2), where a phylogenetic vector |v> is subtracted by the background vector |u_16s_>, the resulting vector |*ε*> is guaranteed to be orthogonal to |u_16s_>. Panel B corresponds to Eq(2'), where a phylogenetic vector |p> is subtracted by the background vector |r>, the resulting vector |p'> may still have residual components along the orientation of |r>. Panel C shows that the resulting vector |p'> may become as nearly orthogonal to |r> when the length of the vector |r> is properly rescaled.

### Super-phylogenetic vectors via TreeSec method

We propose a novel method to utilize the distance matrices and the species tree for predicting protein interactions. There are two major changes to the mirror tree method. First, we augment the phylogenetic vectors with extra bits that encode the topological information of the protein tree with reference to the species tree. Second, in contrast to the unsupervised learning scheme of the mirror tree method, we adopt a supervised learning paradigm, specifically the support vector machines, [20-22] to further tap into the prior knowledge about interacting and non interacting protein pairs. As all proteins are already represented as the phylogenetic vectors of the same dimension, it is convenient to concatenate the two vectors for any pair of proteins and use the concatenated vector to represent the pair. All pairs thus represented are then split into to two subsets – one subset is used for training and the other for testing.

**Figure 2:**
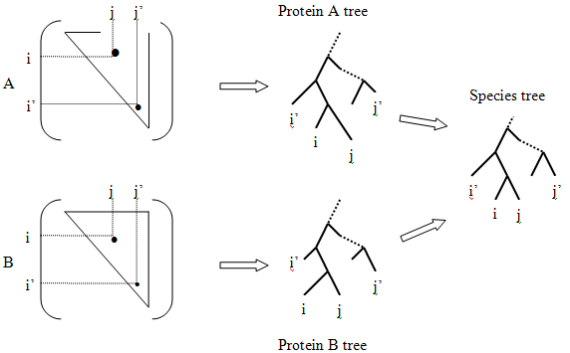
Illustration of how the elements of the distance matrices correspond to distances between leaves on the phylogenetic trees. In matrix A, element (i, j) corresponds to a pair of neighboring genomes, whereas element (i', j') to a pair of genomes that are distantly positioned in the protein A tree, which can be reconstructed from matrix A using the standard methods, such as neighbor-joining algorithm. Likewise, elements (i, j) and (i', j') in matrix B have similar interpretation as corresponding to the respective pairs of genomes in the protein B tree. When comparing two proteins A and B by calculating the Pearson correlation coefficients between the two corresponding matrices, the elements (i, j) and (i', j') should be weighted according to their "importance" dictated by the positions in the trees. It is noted that although the two protein trees shown here have different branch lengths but the same topology, in more complicated cases the tree topologies can also be different. In this study, however, the indices of the two matrices are mapped to the same tree, the species tree. The justification and effect of using the species tree is explained in the text.

The key contribution of our method comes with the data representation, in which we augment the phylogenetic vector, used in both the original and Sato *et al*'s modified version of mirror tree, with some organizational information about the elements in the distance matrix; such information reflects in a somewhat explicit way how the protein tree is reconstructed from the distance matrix, with reference to the species tree. In the mirror tree method, all the elements in the distance matrix are treated equally, as indicated by the un-weighted summation of the product [D_A_(i, j) - Ave(D_A_)] [D_B_(i, j) - Ave(D_B_)] over all (i, j) in calculating the Pearson correlation coefficient as defined in Eq(1). The very rich intra-matrix correlational information is almost entirely neglected and is condensed to just a single number – the average Ave(D_A_), to which the deviation [D_A_(i, j) - Ave(D_A_)] is measured for each element (i, j) and correlated with its counterpart from the other matrix B and then factored into the inter-matrix correlation *ρ*_AB_. For example, if element D_A_(i, j) is above the average in the matrix D_A _and D_B_(i, j) is above the average in matrix D_B_, then the product [D_A_(i, j) - Ave(D_A_)] [D_B_(i, j) - Ave(D_B_)] is positively contributing to the correlation *ρ*_AB_, and thus to the similarity. In another case, if D_A_(i, j) is above the average in the matrix D_A _but D_B_(i, j) is below the average in matrix D_B_, then the product [D_A_(i, j) - Ave(D_A_)] [D_B_(i, j) - Ave(D_B_)] is negatively contributing to the correlation *ρ*_AB_, and thus to the similarity. And each element in the distance matrix is treated independently and equally. However, to a very large degree, it is the intra-matrix correlations among the elements that determine the protein tree, as manifested in the distance based phylogenetic tree reconstruction algorithms such as UPGMA and Neighbor-Joining [20]. For example, as shown in Figure 2, if (i, j) corresponds to two host genomes i and j closely positioned in the tree and (i', j') to a pair of distantly related genomes i' and j', then it makes sense to weight [D_A_(i, j) - Ave(D_A_)] [D_B_(i, j) - Ave(D_B_)] and [D_A_(i', j') - Ave(D_A_)] [D_B_(i', j') - Ave(D_B_)] differently when contributing to the correlation *ρ*_AB _in Eq(1). When measuring matrix similarity, not only may elements in a matrix contribute differently, but the very fact that a tree can be reconstructed out a distance matrix imparts a clear indication of some embedded "intra-matrix" correlations among matrix elements. Therefore it is reasonable to hypothesize that the matrix elements need to be regrouped in a certain way such that the hierarchical relationships among matrix elements can be unraveled and flattened to achieve the effect of "weighted" Pearson correlation between two matrices. This is somehow similar to the ideas in [11-13] where, to predict interaction specificity among paralogous proteins, the rows and columns of the distance matrices are reshuffled in order to find maximal similarity measured as inter-matrix correlation. As the species tree bestows a hierarchy of relationships among the host genomes, weighting the matrix elements in order to reflect the intra-matrix correlations can become very complicated. Here we propose a simple, novel way to account for the intra-matrix correlations.

**Figure 3:**
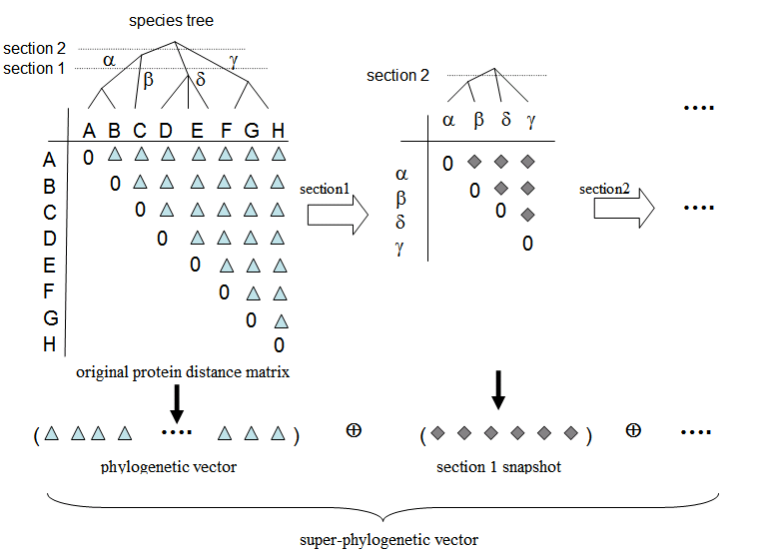
Schematic illustration of TreeSec method to derive super phylogenetic vector from a distance matrix for a given protein from the distance matrix of its orthologous proteins A, B, ..., H. Section 1 across the tree leads to four clusters of the orthologous proteins: *α *= {A, B}, *β *= {C}, *δ *= {D, E, F}, and *γ *= {G, H}. The distances among these clusters are calculated by Eqs. (4–6), resulting in an intermediate matrix. The procedure is repeated for all sections, producing more intermediate matrices. The upper triangles of the matrices are transformed into vectors and concatenated (denoted by the symbol ⊕) into a super phylogenetic vector. In this way, the phylogenetic vector is extended with extra "bits" that encode the topological information of the protein tree with reference to the species tree.

Specifically, we use the species tree (reconstructed by neighbor-joining from a distance matrix of the 16S rRNA sequences of the 41 host genomes) to generate a hierarchical clustering of the genomes, which correspondingly gives a hierarchical clustering of the indices of the protein distance matrices. Given a tree with the root at the top, a "section" cut across the tree will give rise to clusters of leaves, i.e., leaves within the same branch at the section will belong to the same cluster. The number of clusters is equal to the number of branches at the section, and is determined by the tree and the height where the section is cut – the higher the cut is, the fewer the number of clusters is. For example, in Figure 3, section1 generates 4 clusters: *α*, *β*, *γ *and *δ*. Given a protein distance matrix D, for each section, an intermediate distance matrix between all pairs of clusters is derived from the original distance matrix as follows.

D(*α*, *β*) = ∑_i∈C*α*;j∈C*β*_[D(i, j)]/|C_*α*_| |C_*β*_|     (4)

where |C_*α*_| and |C_*β*_| are the size for the clusters C_*α *_and C_*β *_respectively. That is, the distance between two clusters C_*α *_and C_*β *_is equal to the average distance between pairs of orthologous proteins from each cluster. This definition of distance between two clusters is the same as defined in UPGMA during the tree reconstruction [20]. The intermediate distance matrix gives a "snapshot" of the evolutionary history about these orthologous proteins at the time, marked as the tree height, where the section is cut. The snapshot – rather the intermediate matrix derived from it – carries the information about how the hypothetical ancient ancestors at that time are related to one another in terms of evolutionary distance, from the perspective of the protein being studied. Since the matrix is symmetric, only the upper (or lower) triangle is needed, which can be transformed into a vector form and concatenated to the original phylogenetic vector in the mirror tree method. The final representation of a protein is the original phylogenetic vector concatenated with all "snapshot" vectors, which is called *super-phylogenetic vector*. Figure 3 gives a schematic illustration of the procedure for generating the super-phylogenetic vectors. The number of "snapshots" is a free parameter in our method. One simple way to remove this parameter is to use the full spectrum of sections, i.e., having a section made at each branching point in the tree. The difficulty with this full spectrum approach is that the number of sections is large, and many of the neighboring sections are very similar to one another, and therefore not adding much useful information to the phylogenetic vectors; rather it may inflate the dimension of the resulting super-phylogenetic vectors up to 15,000 or higher, which is beyond the capacity of the classifier used in the study. Instead, a value of 6 is used as the number of snapshots taken in the experiments, which yields a dimension of 2386 for super-phylogenetic vector pairs versus the 1640 for the original phylogenetic vector pairs. One refinement made while generating the super-phylogenetic vector is to first adjust the distances, as defined in neighbor-joining algorithm to remove the molecular clock constraint assumed by the UPGMA:

d(i, j) = D(i, j) - (r_i _+ r_j_)     (5)

and

r_i _= ∑_k _D(i, k)/(|L| - 2)     (6)

where |L| is the dimension of the matrix D. Another refinement is to use the same projection procedure introduced in Sato *et al's *modified mirror tree method Eq(2), only now that |v_i_> and |u_16s_> are substituted with the super-phylogenetic vectors. It should be noted that since the background subtraction in *tol-mirrortree *method and Sato *et al*'s method also utilizes the phylogenetic tree, combining the TreeSec procedure and background subtraction may introduce some redundancy.

The use of the species tree instead of individual protein trees for hierarchical clustering has a twofold effect. One effect is more theoretical; it is to reveal how individual protein trees (embedded in the distance matrices) would differ from the underlying species tree of the host genomes, in the same spirit of subtracting the common background as in [9,10]. The other effect is more pragmatic; it ensures that the super-phylogenetic vectors thus obtained have the same dimension for all proteins, and therefore can be readily used as input to the support vector machine.

### SVM

The classifier used here is a support vector machine. As a powerful statistical learning method, support vector machines (SVMs), originally proposed by Vapnik [21,22], have recently been applied with remarkable success in bioinformatics problems, including remote protein homology detection, microarray gene expression analysis, and protein secondary structure prediction [24].

There are a couple of reasons to use SVMs. First, the data are already in the vector form, particularly suitable as inputs for SVMs. Second, SVMs have been used to predict protein-protein interaction in previous works [25,26], though there the different properties of proteins are used. We plan to have a comprehensive study of using SVM on data from different sources, and more importantly, how to combine them for better prediction. Third, SVMs have some inherent advantages over other classifiers, including: 1. quadratic programming to avoid local minima, 2. geometric intuition, 3. lower Vapnik-Chervonenkis dimension leading to better generalization, and 4. amicability with small training samples, which all contribute to its popularity as a classifier adopted in many applications.

The basic idea of SVMs is simple; it is to find a hyperplane that separates two classes of objects, as represented as points in a vector space, with the maximum margin to the boundary lines. Such a hyperplane ensures good generalization and unseen data are then classified according to their location with respect to the hyperplane. The power of SVMs comes partly from the data representation, where an entity, e.g., a pair of proteins, is represented by a set of attributes. However, how those attributes contribute to distinguishing a true positive from a true negative may be quite complex. In other words, the boundary line between the two classes, if depicted in a vector space, can be highly nonlinear. The SVMs method will find a nonlinear mapping that transform the data from the original space, called input space, into a higher dimensional space, called feature space, where the data can be linearly separable.

In general, the mapping can be quite complex and the dimension can be very (even infinitely) high in order for the mapped data to be linearly separable. The trick of SVMs is the use of kernel functions, which define how the dot product between two points in the feature space, which is the only quantity needed to solve the quadratic programming problem for finding the maximum margin hyperplane in the feature space. The use of kernel functions avoids explicit mapping to high dimensional feature space; high dimensionality often poses difficult problems for learning such as over-fitting, thus termed the curse of dimensionality. Polynomial kernel and Gaussian kernel are the two most commonly used generic kernels – linear kernel is not really useful in most cases except when the data are linearly separable. For vectors x and y, Gaussian RBF is defined as

K(x, y) = exp[- (|x-y|^2^/c)],     (7)

and the polynomial kernel is defined as

K(x, y) = [1 + s(x · y)]^d^,     (8)

where c, *s *and *d *are parameters adjustable in the software package SVMLight [27]. Both kernels are experimented with the default values for c, *s *and *d*, and the Gaussian kernel yielded the best results reported in this paper. Because the polynomial kernel performs significantly worse with the default setting, we also tested with changing the polynomial degree d from the default value (d = 3). We found that the performance is quite sensitive to the degree. Details are given in the next section. Besides using the separation of training and testing as a mechanism to alert us to overfitting, another mechanism built into SVMLight for avoiding overfitting is the use of "soft" margin, i.e., to allow for misclassification for some outlier training data points, and a cap on the number of iterations to stop the optimization process during the training even if the preset error rate is not reached. And we have used SVMLight's default setting for our experiments.

It is worth noting that, overall our method can be viewed as a hybrid that employs in tandem both an explicit mapping, from phylogenetic vectors to super-phylogenetic vectors, and the use of a generic kernel.

## Results and discussion

We test our TreeSec method in a series of leave-one-out cross-validation experiments on the data set described above. To prepare an experiment, one of the 13 interacting pairs is selected and reserved as the positive testing example, and 48 non interacting pairs that contain one protein from the positive testing example are reserved as negative testing examples. The rest 325 - 49 = 276 pairs are used as training examples, among which there are 12 interacting pairs as positive training examples. By rotating the positive testing example among the 13 interacting pairs, we can design 13 such leave-one-out cross validation experiments, and the average performance is reported.

**Figure 4:**
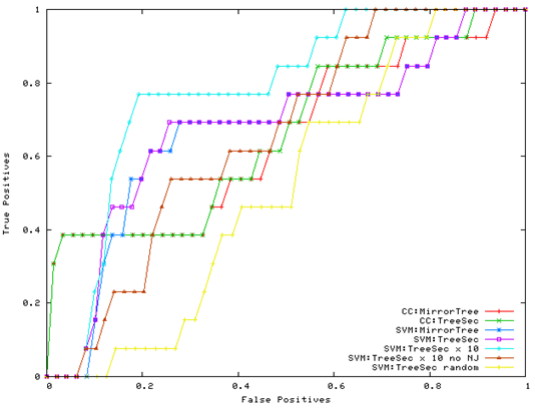
ROC Curves for Predictions using unsupervised learning with Correlation Coefficients and supervised learning with a SVM of Gaussian kernel.

For each experiment, the training examples are taken as input to train a support vector machine. The implementation of the support vector machine is adopted from the SVMLight package [27]. The two commonly used kernel functions – polynomial, and RBF – are experimented with the default parameter settings, and the Gaussian RBF kernel function scored the best performance, which is reported in Table 3. With the SVM trained, the 49 testing examples are then input to it for prediction. A score with real value between -1 and +1 is assigned by the SVM to each testing example. Ideally, a positive score indicates a predicted positive, whereas a negative score indicates a predicted negative. This implies a perfect cutoff score at zero. In practice, the cutoff score may be set at a different value, other than zero. Indeed, its actual value does not matter, as long as the predicted positives (i.e., with a score higher than the cutoff) are true positive, and the predicted negative (i.e., with a score lower than the cutoff) are true negative. To evaluate the performance, we use the receiver optical characteristic (ROC) score, which is the normalized area under a curve that plots the number of true positives as the number of false positives when a moving cutoff score scans from +1 to -1 [28]. The ROC score is 1 for a perfect performance, whereas a random predictor, which will uniformly mix up positives and negatives, is expected to get a ROC score 0.5. Some ROC curves for our experiments are shown in Figure 4.

**Table 3:** ROC scores

**Classification**	**Data Representation**	**ROC**			
Unsupervised (Pearson CC)	MirrorTree	0.6587			
	TreeSec	0.6731			
Gaussian Kernel SVM	MirrorTree	0.7067			
	TreeSec	0.7436			
	TreeSec × 10	0.8446			
	TreeSec × 10 (no NJ)	0.7196			
	TreeSec × 10 (random tree)	0.5368			
	**Polynomial degree**	**2**	**3**	**4**	**5**
Polynomial Kernel SVM	MirrorTree	0.7212	0.5353	0.7003	0.5577
	TreeSec	0.7196	0.5657	0.7179	0.6074
	TreeSec × 10	0.7051	0.6426	0.7468	0.6619
	TreeSec × 10 (no NJ)	0.6987	0.5865	0.6907	0.6554
	TreeSec × 10 (random tree)	0.4696	0.4583	0.4503	0.5048

The ROC scores of the mirror tree method and our TreeSec method, with a few variations, are reported in Table 3. Since the mirrortree is an unsupervised learning method, to be fair, we first use TreeSec in an unsupervised learning manner, and compare the two. In this case, since there is no training necessary, for each leave-one-out experiment, only the testing examples are ranked by their Pearson correlation coefficients as if they were scores output from a classifier. The mirror tree method using the phylogenetic vectors prepared via Sato *et al*'s procedure receives a ROC score 0.6587. A slightly higher ROC score (0.6731) is obtained when everything is kept the same except for substituting the phylogenetic vectors with the super-phylogenetic vectors prepared via TreeSec method.

The advantage of TreeSec method becomes more obvious when used in supervised learning. In this case, TreeSec and MirrorTree are compared for their capability of representing proteins in a way which is more conducive for classification. Once proteins are represented as super-phylogenetic vectors via TreeSec or as phylogenetic vectors via MirrorTree, they are fed into the same classifier, in this case, a SVM. As we see in Table 3, while the performance of the phylogenetic vectors (MirrorTree) also improves (ROC score 0.7212 with a degree 2 polynomial kernel), the super-phylogenetic vectors prepared by TreeSec obtain a significantly better ROC score (0.8446 with a default setting Gaussian kernel). Because of the significantly worse performance for polynomial kernel with a default degree *d *= 3, we tested with changing the degree to different values and found that the performance is significantly better for even values than odd values of *d*. This phenomenon may be an indication of the parity of the hyperplane in the feature space: symmetric with respect to changing the sign of the coordinates. Overall in Table 3 better performance has been noted for "TreeSec × 10" when the "snapshots" are taken at a more spacious interval by multiplying the tree height with a factor of 10. Because the distances obtained from the PHYLIP software are typically small fraction numbers, dividing the distances at the "cutting" points tend to yield rounding errors, and a re-scaling of the distances in the tree to bigger values proved to be helpful with avoiding such a problem. In Table 4, the effect of the re-scaling factor of the learning performance is given, showing that Gaussian kernel is more affected by the rescaling than is the polynomial kernel.

**Table 4:** ROC scores for TreeSec × N

	**N**						
Kernel	2	4	6	8	10	12	14
Gaussian	0.7564	0.7564	0.7612	0.7628	0.8446	0.8205	0.8013
Polynomial (d = 2)	0.6298	0.6298	0.6298	0.6522	0.7051	0.6779	0.6795

The dimension of the super-phylogenetic vectors from TreeSec is obviously higher than that of the phylogenetic vectors in MirrorTree, since the former is derived by concatenating extra bits of information to the latter. Although this may raise concerns with a judicious reader about the fairness for comparing the two approaches if they have different sizes of data, it should not be a problem in our case, because we use the same amount of input data as the Sato *et al*'s approach – the same distance matrices with the same size for proteins and the same distance matrix for species (based on 16S rRNA sequences). The extra bits of information are not really extra; they are the result of how we unravel the information embedded in the input data. In a sense, our method is a hybrid of combining the explicit mapping (to higher dimension) and the use of kernels, which may explain why our method bodes well with the learning task. Nonetheless, care should be taken to not let the increase of dimension go unchecked, as redundancy may arise and lead to overfitting and bad generalization. That is part of the reason that only "snapshots" of evolutionary history are incorporated.

It is not surprising that the neighbor-joining distance adjustment is essential; without it as shown in Table 3 the performance decreases significantly (0.69). To verify that the better performance indeed arises from incorporating the intra-matrix correlations, we run the same experiments on the data prepared using a random species tree, we can see in Table 3 that the ROC scores are consistently low at around 0.5 for cases when a random tree is used.

To further examine the performance, in Figure 4, ROC curves are shown for those ROC scores reported in Table 3 for the unsupervised learning based on Pearson correlation coefficient and for the supervised learning with a Gaussian kernel SVM. Given the Y-axis as the true positives and X-axis as the false positives, the higher a curve means more true positives are identified at cost of a given number of false positives. It is consistent to note that "TreeSec × 10" corresponds to the top curve overall. Also remarkable is the steep slope for the two ROC curves corresponding to the unsupervised learning (CC:MirrorTree and CC:TreeSec) at small false positive rates (X < 0.1). This explains the high specificity for these unsupervised learning based on correlation coefficient, and is consistent with what is reported in [9,10]. As moving to the right (i.e., when more false positives are made), these two curves quickly lose the upward momentum (i.e., identify fewer true positives), an indication of low sensitivity. Therefore, the supervised learning using the SVMs in these experiments offers a better balance between sensitivity and specificity.

It is worth noting that, the highly skewed learning problem is likely a reflection of situations in the real world, i.e., there are far more negatives than positives. In our case, given n proteins that uniquely interact with only one other member, there are only n/2 positive pairings among the (n^2 ^- n)/2 possible pairings of these n proteins. That is, the interacting network, with nodes representing the proteins and edges representing the interactions, is quite sparse, but our method is still applicable when there are more edges. Because every possible pair of nodes is assigned a score in our method, predictions can be made by going down a list of all pairs that are ranked by their scores in decreasing order. So, regardless the number of the actual edges in the network (sparse or not), the method works, and perfectly so as long as the true interacting pairs are ranked higher than non interacting pairs in the prediction. Indeed, this scheme is also widely used in predicting protein interaction networks in general, both in an unsupervised paradigm such as the original mirror tree method, and in a supervised learning paradigm.

## Conclusion

To summarize, in this work we developed a novel, simple method to explore the intra-matrix correlational information embedded in the distance matrices and incorporate such information into a data representation which is conducive to supervised learning. Three methods recognize the importance of the phylogenetic tree, both Sato *et al*'s projection method [9] and Pazos *et al*'s *tol-mirrortree *[10] try to "subtract" from the similarity (measured as correlation coefficients) the effect due to speciation rather than the interaction pressures, whereas our method seeks to "unravel" the intrinsic structure of the distance matrices using the species tree as a guide and then "concatenate" these snapshots of the evolutionary history to the current view (i.e., the original ortholog distance matrices) of the proteins. That is, the main difference between subtracting and adding is that the former is more appropriate for removing background noise so as to reduce false positive and the latter is more appropriate for disentangling intra-matrix correlations so as to aid a supervised learner.

As future work, we will study how the reconciliation between protein trees and species tree can be more explicitly represented and how to associate selection pressure imposed by the interaction to specific evolutionary events, e.g., horizontal gene transfers.

## Competing interests

The author(s) declare that they have no competing interests.

## Authors' contributions

LL conceived the ideas and prototyped some of the implementation, and RAC collected the data and implemented the methods. Both authors participated in writing the manuscript.
